# Thermo-Sensitive Microgel/Poly(ether sulfone) Composited Ultrafiltration Membranes

**DOI:** 10.3390/ma16145149

**Published:** 2023-07-21

**Authors:** Wei Fan, Shaoxiong Zhu, Jingjing Nie, Binyang Du

**Affiliations:** 1 State Key Laboratory of Motor Vehicle Biofuel Technology, Department of Polymer Science & Engineering, Zhejiang University, Hangzhou 310027, China; 22029010@zju.edu.cn (W.F.); sxzhu@zju.edu.cn (S.Z.); 2 Department of Chemistry, Zhejiang University, Hangzhou 310027, China; niejj@zju.edu.cn

**Keywords:** microgels, ultrafiltration, membrane, thermo-sensitivity

## Abstract

Thermo-sensitive microgels known as PMO-MGs were synthesized via surfactant free emulsion polymerization, with poly(ethylene glycol) methacrylate (OEGMA_475_) and 2-(2-methoxyethoxy) ethyl methacrylate (MEO_2_MA) used as the monomers and N, N-methylene-bis-acrylamide used as the crosslinker. PMO-MGs are spherical in shape and have an average diameter of 323 ± 12 nm, as determined via transmission electron microscopy. PMO-MGs/poly (ether sulfone) (PES) composited ultrafiltration membranes were then successfully prepared via the non-solvent-induced phase separation (NIPS) method using a PMO-MG and PES mixed solution as the casting solution. The obtained membranes were systematically characterized via combined X-ray photoelectron spectroscopy, field-emission scanning electron microscopy, Fourier transform infrared spectroscopy and contact angle goniometer techniques. It was found that the presence of PMO-MGs significantly improved the surface hydrophilicity and antifouling performance of the obtained membranes and the PMO-MGs mainly located on the channel surface of the membranes. At 20 °C, the pure water flux increased from 217.6 L·m^−2^·h^−1^ for pure PES membrane (M00) to 369.7 L·m^−2^·h^−1^ for PMO-MGs/PES composited membrane (M20) fabricated using the casting solution with 20-weight by percentage microgels. The incorporation of PMO-MGs also gave the composited membranes a thermo-sensitive character. When the temperature increased from 20 to 45 °C, the pure water flux of M20 membrane was enhanced from 369.7 to 618.7 L·m^−2^·h^−1^.

## 1. Introduction

Since the late 1960s, membrane separation technologies have been widely used in chemical processes, environmental protection, biopharmaceutical, food safety, water purification and other aspects of production because of their advantages, such as low energy consumption [1], short operation time, high safety and good environmental compatibility [2]. Energy and environmental issues are major difficulties that face modern civilization, and membrane separation technologies offer a viable solution, which is in line with the need for further economic and social development. Organic polymers have excellent membrane-forming properties, chemical stability, thermal stability and acid and alkali resistance. Therefore, polymers have become the most widely studied and used membrane materials. The polymer materials most commonly used in the production of separation membranes are polyethylene (PE), polypropylene (PP), poly(vinylidene fluoride) (PVDF), polysulfone (PSF) and poly(ether sulfone) (PES) [3,4,5]. However, the hydrophobicity of polymer separation membranes makes it easy for them to adsorb pollutants during the separation process, causing membrane pollution and a decline in water flux [6,7,8]. As a result, modification of polymer membranes to improve their permeability, hydrophilicity, separation and antifouling properties becomes a long-term topic of interest in the field of polymer separation membranes [9,10]. The methods used for modification of polymer membranes include physical and chemical methods. Surface coating [11] and blending modification [12] of polymer membranes are physical methods. Grafting functional polymer onto the surface of a separation membrane is a chemical method [13,14,15]. Blending a polymer that contains special functional groups with membrane-forming polymers is an economical and efficient one-step modification method for modifying polymer membranes that has been widely used in industry due to its straightforward procedure and reasonable cost [16].

Environmentally responsive separation membranes can be fabricated by combining the stimulus-responsive materials with the separation membrane via chemical or physical methods, which may be response to environmental stimuli, like temperature or pH [17,18,19]. The stimulus-responsive materials serve as intelligent switches that control the obtained separation membrane, adjusting the membrane’s performance based on their response to the external stimuli [20,21]. Among them, temperature responsive materials are frequently employed to fabricate thermo-sensitive separation membranes due to their simple temperature control properties [22,23,24]. The change in temperature will affect the hydrophilic and hydrophobic properties of the corresponding temperature-responsive materials [25,26]. As a result, the pore size and surface hydrophilicity of the resultant membranes could be controlled by changing the temperature [27,28]. For example, Wu et al. [29] fabricated thermo-sensitive membranes by grafting poly(*N*-isopropylacrylamide-*co*-glycidyl methacrylate) (PNG) thermo-responsive microgels to the aminated poly(ethylene terephthalate) (PET) membranes, and they found that the water flux of the obtained membranes altered abruptly when their temperature reached the volume transition temperature (VTT) of the PNG microgels. Xie et al. [30] used the vapor-induced phase separation (VIPS) method to prepare ethanol-responsive PVDF membranes using poly(*N*-isopropylacrylamide) (PNIPAM) microgels as intelligent switches, and the obtained membranes showed ethanol- and thermo-responsive characteristics.

In this work, thermo-sensitive microgels known as PMO-MGs were synthesized via surfactant free emulsion polymerization (SFEP), with poly (ethylene glycol) methacrylate (OEGMA_475_) and 2-(2-methoxyethoxy) ethyl methacrylate (MEO_2_MA) used as the monomers and *N*, *N*-methylene-bis-acrylamide (BIS) used as the crosslinker. The pure PES ultrafiltration membrane was hydrophobic, and its surface was easily subjected to protein contamination during ultrafiltration operation. The incorporation of hydrophilic thermo-sensitive microgels into PES ultrafiltration membranes might improve the hydrophilicity of the membrane surface, hence enhancing the antifouling performance of the composited membranes. Furthermore, the pore size of resultant composited membranes could be adjusted by changing the operating temperature, leading to improved thermo-adjustable ultrafiltration performance. The obtained PMO-MGs were mixed with PES via the physical blending method to form the casting solutions, which were then used to fabricate thermo-sensitive PMO-MGs/PES composited ultrafiltration membranes via the nonsolvent-induced phase separation (NIPS) method. The oligomer of MEO_2_MA had a low critical solution temperature (LCST) of about 20 °C, and the oligomer of OEGMA_475_ had a LCST of about 90 °C [31,32,33]. By adjusting the ratio of MEO_2_MA and OEGMA_475_, a thermo-sensitive PMO-MGs with VTT in the range of 20–45 °C could be obtained [34,35,36]. PMO-MGs have excellent biocompatibility [37,38,39], making them suitable for application in the fields of medicine, biology and separation membranes [40,41,42]. The effects of PMO-MGs on the morphology, porosity, surface hydrophilicity, ultrafiltration properties, antifouling performance and temperature responsibility of the obtained PMO-MGs/PES composited ultrafiltration membranes were systematically investigated and discussed. Compared to the traditional PES membranes, the obtained composite membranes had improved hydrophilicity, anti-contamination properties, water permeability and responsiveness to the change in external temperature.

## 2. Materials and Methods

### 2.1. Materials

Poly(ether sulfone) (PES, Ultrason E6020, Mw = 58,000) samples were obtained from BASF, Shanghai, China. *N*-Methyl pyrrolidone (NMP, 98%) and potassium persulfate (KPS) were purchased from Macklin Biochemical Co., Shanghai, China. Poly(ethylene glycol) methacrylate (OEGMA_475_, Mn = 475, 98%) and bovine serum albumin (BSA, Biotech grade, 96%) were purchased from Aladdin Chemical Ltd., Shanghai, China. 2-(2-methoxyethoxy) ethyl methacrylate (MEO_2_MA, 99%) was purchased from J&K Chemical Ltd., Shanghai, China. *N*, *N*-methylene-bis-acrylamide (BIS, 96%) was purchased from Thermo Fisher Scientific, Shanghai, China. All chemicals were used as received. Ultrapure water was created in a laboratory.

### 2.2. Synthesis of PMO-MGs

PMO-MGs were synthesized via SFEP. MEO_2_MA (9.5 mmol), OEGMA_475_ (0.5 mmol) and BIS (0.6 mmol) were dissolved in ultrapure water (49 mL) in a three-necked flask at 70 °C. The mixture was agitated magnetically in a nitrogen environment for 60 min. To start the copolymerization process, 1 mL of an aqueous solution that contained 30 mg of KPS was added to the mixture. For an additional 6 h, the process was kept at 70 °C. After polymerization, milky white microgel suspension was obtained, which was then cooled to room temperature and purified via dialysis in ultrapure water with a molecular weight cutoff (MWCO) of 1000 for 3 days. The ultrapure water was replaced every 8 h. Finally, the obtained purified microgels were freeze-dried.

### 2.3. Characterization

Using Fourier transform infrared spectroscopy, PMO-MGs’ chemical structure was characterized (330FT-IR, Thermo Nicolet Corporation, Madison, WI, USA). PMO-MGs’ hydrodynamic diameter was determined using dynamic light scattering (DLS) and a 90 Plus Particle Analyzer (Brookhaven Instruments Corp., Holtsville, NY, USA). The morphology of PMO-MGs was detected by using a HT-7700 transmission electron microscopy (Hitachi, Tokyo, Japan). The change in transmittance of PMO-MGs’ suspensions as a function of temperature was recorded using a Cary 100 UV-Vis spectrophotometer (Varian Australia Pty Ltd., Victoria, Australia).

The surface properties and composition of the obtained PMO-MGs/PES composited ultrafiltration membranes were characterized via attenuated total reflection Fourier transform infrared spectroscopy (330FT-IR, Thermo Nicolet Corporation, Madison, WI, USA) and X-ray photoelectron spectroscopy (K-Alpha, Thermo Scientific, Waltham, MA, USA), with Al Kα used as a radiation source. The morphology of the membranes’ upper surface and cross-section were investigated using scanning electron microscopy (S-4800, Hitachi, Tokyo, Japan). Membranes’ cross-sections were obtained via quenching the membranes in liquid nitrogen. The thickness of membrane was obtained by calculating the cross-section size using ImageJ. Prior to SEM observation, the surfaces of the membranes were sputtered with gold for 30 s via physical vapor deposition. The concentrations of BSA solution were determined via a Cary 100 UV-Vis spectrophotometer (Varian Australia Pty Ltd., Mulgrave, Victoria, Australia).

### 2.4. Preparation of PMO-MGs/PES Composited Ultrafiltration Membranes

PMO-MGs/PES composited ultrafiltration membranes with different microgel contents were prepared via the NIPS method. The following description of the preparation process was given:

Firstly, the freeze-dried PMO-MGs were uniformly dispersed in NMP (100 g) under ultrasonication for 4 h. Subsequently, 18 g of PES was added. To create a homogenous solution, the combined solution was mechanically agitated at 70 °C for 24 h. To release the bubble, the combined solution was allowed to rest for 24 h. The detailed compositions of mixed solutions are summarized in Table 1. The value of MG/PES was the mass ratio of PMO-MGs to PES.

The mixed solution was then cast, which was performed by employing the casting knife with a gate height of 250 μm on a clean glass plate. The glass plate and moist membrane were placed in the ultrapure water at room temperature. Following a thorough separation of the primary membrane from the substrate, they were washed with ultrapure water and kept in the ultrapure water for further use. The membranes were kept clean by changing the ultrapure water every 6 h, which also helped to remove any remaining solvent. The obtained PMO-MGs/PES composited ultrafiltration membranes were coded as MXX, with the numeric number of XX representing the mass ratio of PMO-MGs to PES, as shown in Table 1.

### 2.5. Contact Angle Measurement of PMO-MGs/PES Composited Ultrafiltration Membranes

By applying a contact angle goniometer (OSA200-E, Ningbo NB Scientific Instruments Co., Ltd., Ningbo, China), the surface hydrophilicity of the membrane was quantified via dynamic contact angles. Before measurement took place, the membrane was attached to a glass slide and allowed to dry for 12 h in a vacuum oven. A water droplet (approximately 2.0 μL) was deposited onto the upper surface of the membrane at ambient temperature. It took 60 s to capture the way in which the membrane’s contact angle changed over time.

### 2.6. Membrane Porosity and Pore Size Measurement

The porosity and pore size of the obtained PMO-MGs/PES composited ultrafiltration membranes were calculated using the methods described in the literature [43]. Firstly, the membranes were taken out of the ultrapure water. The water droplets on the membrane surfaces were then dried using tissue papers. The wet weights of the membranes were obtained by weighing the membranes. Afterwards, the membranes were dried at 50 °C and weighed again to give the weight of the dry membrane. The porosity (*ε*) was determined using Equation (1)

(1) $$\varepsilon =\frac{m_{w}-m_{d}}{A\times l\times \rho }\times 100\%$$

where *m_w_* (g) was the membrane’s wet weight, *m_d_* (g) was the weight of the dry membrane, *ρ* (g/cm^3^) was the density of the water, *A* (cm^2^) was the membrane’s area, and *l* (cm) was the membrane’s thickness.

Equation (2) was used to determine the membrane’s average pore size (*r_m_*)

(2) $$r_{m}=\sqrt{\frac{(2.9-1.75\varepsilon )\times 8\eta lQ}{\varepsilon \times A\times \Delta P}}$$

where *r_m_* (nm) was the average pore size, *ε* was the porosity of the membrane, *Q* (m^3^/s) was the pure water flux of the membrane, *η* was the viscosity of the water at given temperature, Δ*P* was the water pressure used for testing, *A* (m^2^) was the contact area of the membrane, and *l* (m) was the membrane’s thickness.

The average result obtained via three measurements served as the membranes’ porosity and average pore size.

### 2.7. Performance of PMO-MGs/PES Composited Ultrafiltration Membranes

#### 2.7.1. Water Flux of the Membranes

The volume of water that flowed through the PMO-MGs/PES composited ultra-filtration membranes per unit of time and area under a certain test pressure was known as the pure water flux [44,45]. Utilizing a cross-flow membrane filtration apparatus with a constant trans-membrane pressure of 0.2 MPa, the filtration tests were conducted. The contact area of PMO-MGs/PES composited ultrafiltration membrane was a circle that had a diameter of 30 mm.

The membranes were pre-pressurized using ultrapure water under 0.25 MPa for 1 h before measurement in order to achieve steady flux. The membrane’s temperature-sensitive water flow was tested at 0.2 MPa in the 20–45 °C range with a 5 °C step increase. For each measured temperature, the water was passed through the membrane for 1 h to ensure that the membrane reached the corresponding temperature. Afterward, the water flux of the membrane was tested for 5 min. Three measurements were made, and the average value was taken and presented.

The water flux (*J_w_*) was calculated via Equation (3):

(3) $$J_{w}=\frac{Q}{A\times t}$$

where *Q*, *A* and *t* were the permeate volume (L) of the water solution, the effective contact area of the membrane (m^2^) and the permeation time (h), respectively.

#### 2.7.2. BSA Rejection Property of the Membranes

The rejection ability of PMO-MGs/PES composited ultrafiltration membranes to BSA solution was tested. The permeation of BSA solution through the membrane was recorded in the 20–45 °C temperature range and using the same instrument and operating conditions. At a pressure of 0.2 MPa for 1 h, the BSA aqueous solution (1.0 mg/mL) penetrated through the membrane. The BSA rejection rate *R* was calculated via Equation (4):

(4) $$R=(1-\frac{C_{p}}{C_{f}})\times 100\%$$

where *C_p_* and *C_f_* were the concentration of the permeated and feeding BSA solutions, respectively, which were determined based on the absorbance at a wavelength of 280 nm. The average value of the three measurements was presented.

#### 2.7.3. Antifouling Performance of the Membranes

The antifouling performance of the PMO-MGs/PES composited ultrafiltration membranes was investigated using ultrapure water and 1.0 mg/mL of BSA solution at 0.2 MPa and 25 °C. The membrane was pre-pressurized using ultrapure water at a pressure of 0.25 MPa for 1 h prior to testing, meaning that the measured membrane could reach a steady flux. Afterward, the water flux of the membrane was recorded at 0.2 MPa. The initial pure water flux *J_w_*_1_ of the membrane was measured. Subsequently, the ultrapure water was changed into a BSA aqueous solution, which was used as the feeding solution, and the permeability flux of BSA aqueous solution *J_BSA_* was measured at 0.2 MPa. The membrane was then cleaned using ultrapure water for 1 h, and the pure water flux *J_w_*_2_ of the membrane was measured again at 0.2 MPa. The flux recovery ratio (*F_R_*) was then determined via Equation (5).

(5) $$F_{R}=\frac{J_{w2}}{J_{w1}}$$

Furthermore, three parameters were also used to quantify the membranes’ resistance to fouling [46]. The reversible fouling ratio (*R_r_*) showed that the development of the filter cake layer reduced flux. As a result of hole plugging and the increase in pollutants on the membrane surface or pores, the flux was irreversibly reduced, as shown based on changes in the irreversible fouling ratio (*R_ir_*). The total fouling ratio (*R_t_*) described the overall flux reduction in the membrane after the contamination of protein. The three fouling resistance ratios were calculated as follows:

(6) $$R_{r}=\frac{J_{w2}-J_{BSA}}{J_{w1}}\times 100\%$$

(7) $$R_{ir}=\frac{J_{w1}-J_{w2}}{J_{w1}}\times 100\%$$

(8) $$R_{t}=R_{r}+R_{ir}$$

## 3. Results and Discussion

### 3.1. Synthesis and Characterization of PMOmicrogels

**Figure 1:**
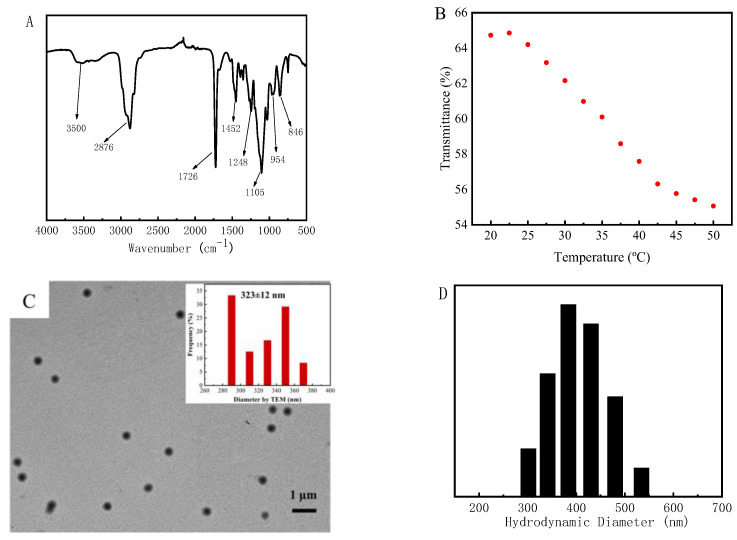
(**A**) The FTIR spectrum of PMO-MGs. (**B**) The light transmittance of PMO-MGs suspension with a concentration of 1.2 mg/mL as a function of the temperature measured via a UV-Vis spectrophotometer at wavelength of 500 nm. (**C**) The representative TEM image of PMO-MGs and the relative size distribution of PMO-MGs. (**D**) The hydrodynamic diameter of PMO-MGs in aqueous suspensions via DLS at 25 °C.

PMO-MGs were synthesized via SFEP, with MEO_2_MA and OEGMA_475_ used as the monomers and BIS used as the crosslinker. The FTIR spectrum of PMO-MGs was shown in Figure 1A. The broad peak at around 3500 cm^−1^ was due to water absorption by PMO-MGs. The absorption bands at 2876 cm^−1^ and 1452 cm^−1^, which were the characteristic peaks of stretching and bending vibration of C-H groups, respectively. Furthermore, the absorption bands at 1726 cm^−1^, 1248 cm^−1^ and 1105 cm^−1^ were ascribed to the stretching vibrations of the C=O, C-C and C-O-C groups, respectively. The absorption peaks at 954 cm^−1^ and 846 cm^−1^ were the bending vibration peaks of C-O-C group. There was no absorption band at 1650 cm^−1^, which was characteristic of C=C. The FTIR results indicated that the successful copolymerization of the monomers and crosslinker occurred, leading to the formation of microgels.

Figure 1B shows the light transmittance of PMO-MGs l suspensions as a function of temperature. The light transmittance of the microgel gradually decreased when the temperature rose from 20 to 45 °C, indicating that PMO-MGs were thermo-sensitive. The volume transition temperature could be determined via the first derivative of the transmittance–temperature curve to be about 36.2 °C. The representative TEM image of PMO-MGs is shown in Figure 1C. PMO-MGs exhibited spherical morphology with uniform size. According to the inset of Figure 1C, the average diameter of PMO-MGs measured using TEM images was about 323 ± 12 nm.

The hydrodynamic diameter of PMO-MGs obtained via DLS at 25 °C was about 403 nm, which was larger than the diameter measured via the TEM picture. This result was understandable because the size of PMO-MGs in a swollen state was larger than that in a dried state, as determined via TEM.

The obtained PMO-MGs were then freeze-dried and used to prepare PMO-MGs/PES composited ultrafiltration membranes, as described in the experimental section.

### 3.2. Microstructures of PMO-MGs/PES Composited Ultrafiltration Membranes

**Figure 2:**
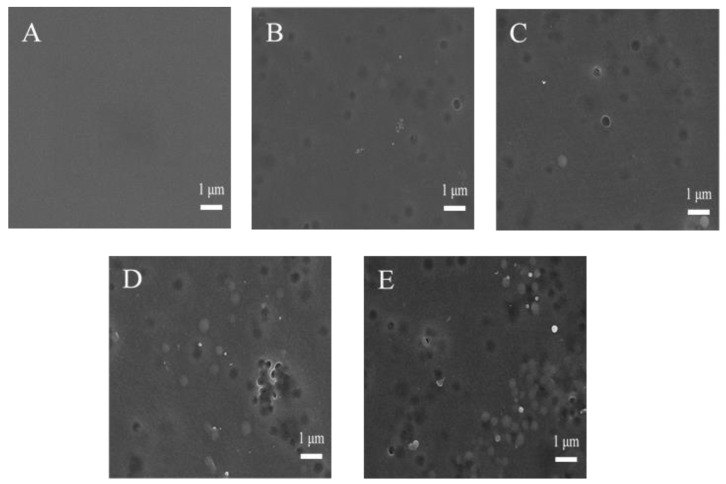
SEM images of the top surface of M00 (**A**), M05 (**B**), M10 (**C**), M15 (**D**) and M20 (**E**) ultrafiltration membranes.

The surface morphology and structure of the M00, M05, M10, M15 and M20 ultrafiltration membranes observed via SEM are depicted in Figure 2. The top surface of the M00 membrane exhibited a dense and smooth structure. For the M05, M10, M15 and M20 ultrafiltration membranes, micropores were observed on the membrane surface. The appearance of micropores was mainly attributed to the hydrophilic characteristic of PMO-MGs, making them spontaneously move to the surface of membranes during the process of NIPS. PMO-MGs located on the upper surface of the membranes were clearly visible. Some PMO-MGs were even released from the polymer-rich phase into the coagulation bath (ultrapure water), causing the surface of the resultant membrane to develop micropores. As the amount of PMO-MGs in the casting solution increased, the number of PMO-MGs and micropores on the membranes’ surface increased. PMO-MGs on the membrane surface had a diameter of around 300 nm, which was similar to the diameter of those observed via TEM. It is understandable that the hydrophilic PMO-MGs migrated from the membrane-forming solution to the interface between the water and polymer phases during the NIPS process, as this process lowered the interface energy [47,48]. As a result, PMO-MGs and micropores were detected on the upper surface of the obtained PMO-MGs/PES composited ultrafiltration membranes.

**Figure 3:**
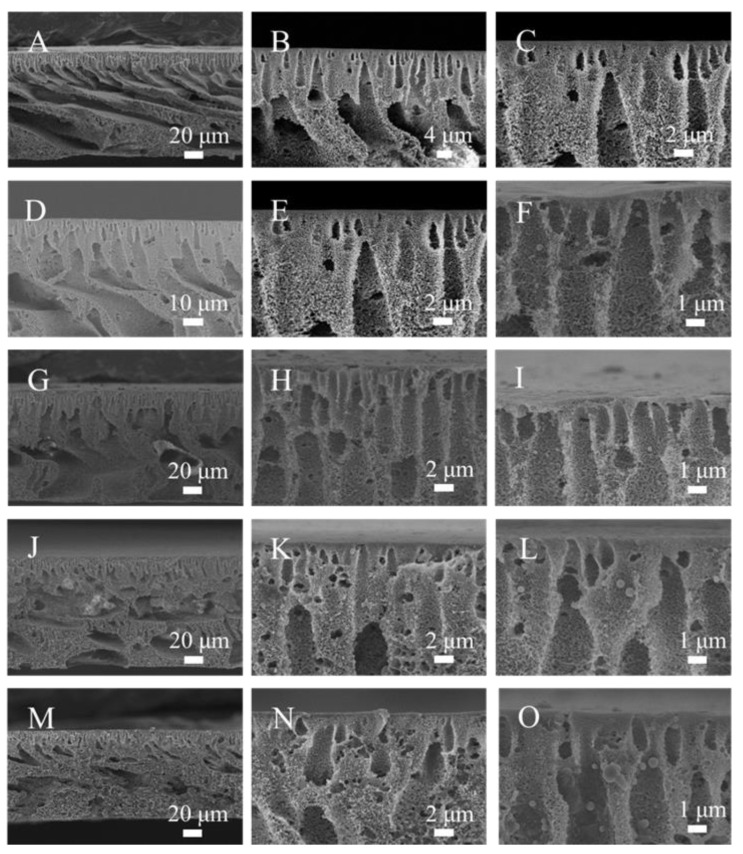
SEM images of the cross-section of M00 (**A**–**C**), M05 (**D**–**F**), M10 (**G**–**I**), M15 (**J**–**L**) and M20 (**M**–**O**) ultrafiltration membranes.

Figure 3 shows the cross-section of the M00, M05, M10, M15 and M20 ultrafiltration membranes with different magnifications via SEM. All of the membranes exhibited typical asymmetric membrane structures. The top section of the membrane had a dense epidermal layer as a selective barrier. The middle and bottom sections of the membrane were composed of finger-like and spongy holes, which gave the membrane its mechanical strength [49]. The channel wall of the M00 ultrafiltration membrane was continuous and smooth, and there were few pores. Compared to the M00 ultrafiltration membrane, the finger-like holes of the M05, M10, M15 and M20 ultrafiltration membranes became wider, which, theoretically, made them more conducive to the passage of water, meaning that the pure water flux of these membranes would be enhanced. During the NIPS process, the top dense layer of the membranes was formed as a result of the exchange in NMP and water. Subsequently, the dense layer reduced the exchange rate of water and NMP, causing the development of a finger-like pore structure [50]. However, the presence of hydrophilic PMO-MGs in the casting solution enhanced the thermodynamic and kinetic instability of casting solution during the NIPS process [30,51]. When microphase separation occurred in the casting solution, the nucleation and growth mechanism state that thermodynamic instability was crucial to the development of microporous structures [52,53]. The presence of hydrophilic PMO-MGs accelerated the phase conversion rate and formed large pores. The enlarged views showed that PMO-MGs were located on the channel surface of the membranes. The number of micropores grew as the PMO-MGs content increased.

### 3.3. Surface Chemical Composition of PMO-MGs/PES Composited Ultrafiltration Membranes

**Figure 4:**
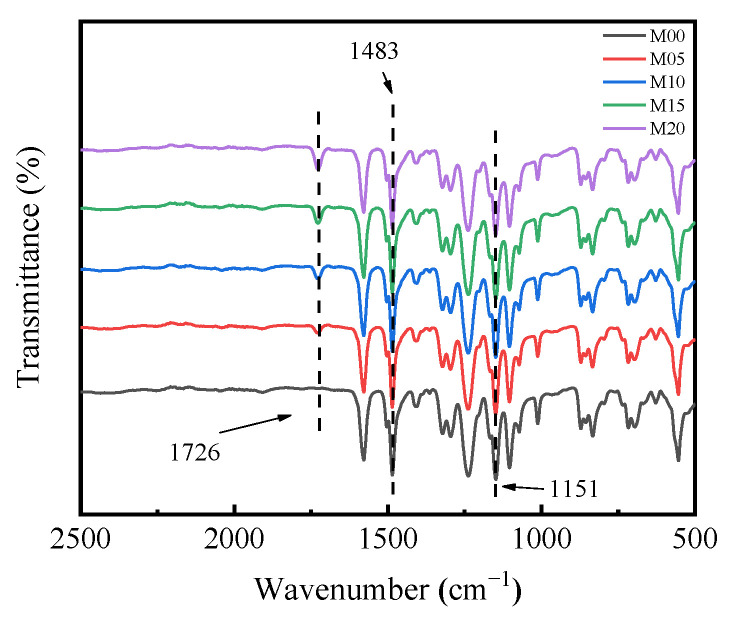
ATR-FTIR spectra of M00, M05, M10, M15 and M20 ultrafiltration membranes.

The PMO-MGs/PES composited ultrafiltration membranes were prepared via the NIPS method, with mixed solutions of PMO-MGs and PES used as casting solutions. The surface chemical composition of the obtained PMO-MGs/PES composited ultrafiltration membranes known as the M00, M05, M10, M15 and M20 were first characterized using ATR-FTIR measurements. Figure 4 shows the ATR-FTIR spectra of different ultrafiltration membranes. We noted that the M00 ultrafiltration membrane represented the pure PES ultrafiltration membrane tested without addition of PMO-MGs. The ATR-FTIR spectrum of the M00 ultrafiltration membrane showed two strong characteristic peaks at 1483 cm^−1^ and 1151 cm^−1^, which were attributed to skeletal vibrations of the O=S=O and benzene ring, respectively. The ATR-FTIR spectra of composited ultrafiltration membranes showed an absorption peak at 1726 cm^−1^, which was the characteristic peak of C=O groups in PMO-MGs. These results confirmed the existence of PMO-MGs on the surface of the PMO-MGs/PES composited ultrafiltration membranes. Furthermore, the intensity of absorption peaks at 1726 cm^−1^ gradually increased between the M05 and M20 membranes, demonstrating that the surface of the resulting composited ultrafiltration had more microgels as the microgel content in the corresponding casting solutions increased.

**Figure 5:**
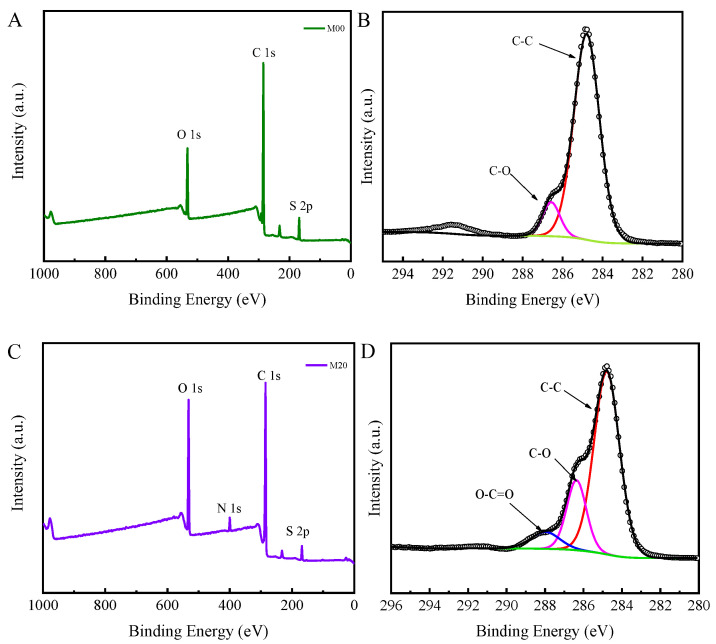
XPS and C1s core-level spectra of M00 (**A**,**B**) and M20 (**C**,**D**) ultrafiltration membranes.

Figure 5 illustrates how XPS was used to further evaluate the surface chemical compositions of the M00 and M20 ultrafiltration membranes. Three peaks at the binding energy levels of 168.2, 285.0 and 532.4 eV could be surveyed using the wide-scan spectrum of the M00 ultrafiltration membrane (Figure 5A), which were assigned to the S2p, C1s and O1s regions, respectively. C1s core-level spectrum of the M00 ultrafiltration membrane included two peaks at 284.8 and 286.4 eV (Figure 5B), which were associated with the benzene and C-O groups of PES, respectively. For the M20 ultrafiltration membrane, XPS wide-scan spectrum exhibited a new peak at 399.9 eV (Figure 5C), which was attributed to N1s region. The presence of N element confirmed the existence of PMO-MGs on the membrane’s surface. The C1s core-level spectrum of the M20 ultrafiltration membrane also showed that a new peak appeared at 288.0 eV, which represented the O-C=O group of PMO-MGs. According to the XPS results, PMO microgels were present on the surface of the M20 ultrafiltration membrane.

Table 2 summarized the surface atomic concentration and atomic ration of the M00 and M20 ultrafiltration membranes. With the incorporation of PMO-MGs, the surface contents of the O and N elements of the resulting PMO-MGs/PES composited ultrafiltration membrane increased. Compared to the M00 ultrafiltration membrane, the content of O element in the M20 ultrafiltration membrane increased significantly from 15.16 to 21.99%. The O/C ratio changed from 0.192 to 0.307. Moreover, the content of N element in the M20 ultrafiltration membrane increased from 0 to 2.99%, and the N/C ratio was improved from 0 to 0.042.

### 3.4. Porosity and Pore Size of PMO-MGs/PES Composited Ultrafiltration Membranes

The porosity and pore size of the M00, M05, M10, M15 and M20 ultrafiltration membranes were determined based on the method described in the experimental section and summarized in Table 3. Compared to the M00 ultrafiltration membrane, the porosity of the M10, M15 and M20 ultrafiltration membranes decreased upon increasing the content of PMO-MGs in the casting solutions. The pore size of the M10, M15 and M20 ultrafiltration membranes increased upon increasing the microgel content in the membranes. The presence of microgels enhanced the thermodynamic and kinetic instability of the casting solution and accelerated the phase transformation process, which was conducive to the expansion of the pore size and enhancement of the interpore connectivity [54]. However, there was a sudden increase in porosity and decrease in pore size for the M05 ultrafiltration membrane compared to those of the M00 ultrafiltration membranes when a small amount of PMO-MG was incorporated into the casting solution. Currently, we do not have a clear explanation for this phenomenon’s occurrence.

### 3.5. Surface Hydrophilicity of PMO-MGs/PES Composited Ultrafiltration Membranes

**Figure 6:**
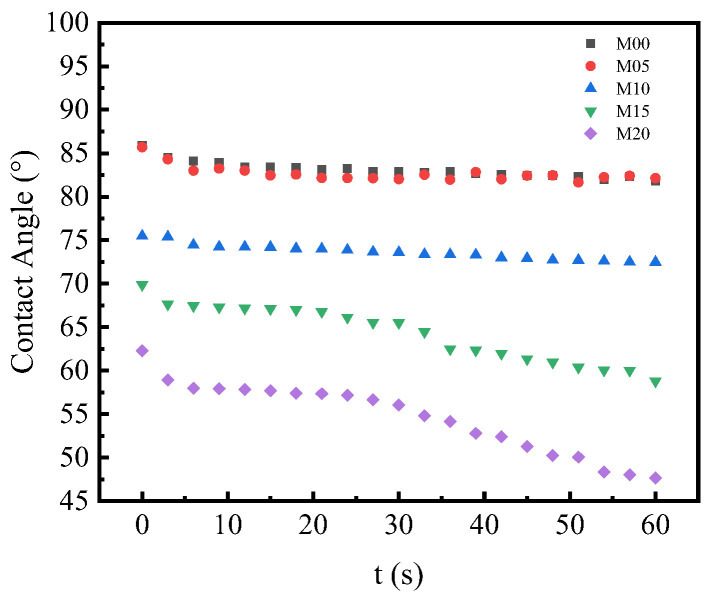
The dynamic water contact angle of M00, M05, M10, M15 and M20 ultrafiltration membranes as a function of time.

The surface hydrophilicity of the obtained PMO-MGs/PES composited ultrafiltration membranes was analyzed by measuring the dynamic contact angle of the membranes. A smaller contact angle and faster water droplet diffusion rate indicated that the surface hydrophilicity of the membrane was high [3]. Figure 6 depicts the dynamic contact angle of the M00, M05, M10, M15 and M20 ultrafiltration membranes as a function of time. The M05 ultrafiltration membrane’s contact angle was comparable to that of the M00 membrane. However, for the M10, M15 and M20 ultrafiltration membranes, the contact angle significantly decreased, indicating that the surface hydrophilicity of the membranes was enhanced by incorporating certain amounts of PMO-MGs. Furthermore, for the M10, M15 and M20 ultrafiltration membranes, the decrease in the contact angle with time was significantly accelerated by the increase in microgel content. These outcomes showed that the surface hydrophilicity of PMO-MGs/PES composited ultrafiltration membranes was enhanced by increasing the microgel content.

### 3.6. Thermo-Sensitive Water Flux of PMO-MGs/PES Composited Ultrafiltration Membranes

**Figure 7:**
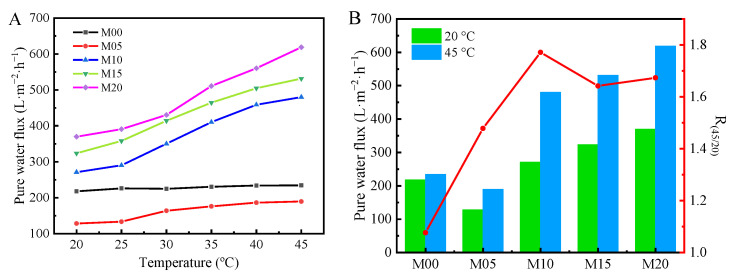
(**A**) Pure water flux of M00, M05, M10, M15 and M20 ultrafiltration membranes as a function of temperature, with water pressure set at 0.2 MPa. (**B**) The thermo-sensitive coefficient R_45/20_ of the corresponding membranes. R_45/20_ was defined as the ratio of pure water flux of the membranes at 45 and 20 °C.

The pure water flux of PMO-MGs/PES composited ultrafiltration membranes was measured at various temperatures with 0.2 MPa. Figure 7A shows the pure water flux of the M00, M05, M10, M15 and M20 ultrafiltration membranes as a function of the temperature. At 20 °C, the pure water flux figures for the M00, M05, M10, M15 and M20 ultrafiltration membranes were 217.6, 128.2, 271.0, 323.6 and 369.7 L·m^−2^·h^−1^, respectively. For the M05 ultrafiltration membrane with microgel content of 5%, the pore size of the resulting membrane decreased to 12 nm, as shown in Table 3. As a result, the pure water flux of the M05 ultrafiltration membrane was even smaller than that of the M00 ultrafiltration membrane without the presence of microgels. However, for the M10, M15 and M20 ultrafiltration membranes with higher microgel content, the pore size of the membranes increased, meaning that the pure water flux significantly increased from 271.0 L·m^−2^·h^−1^ for the M10 ultrafiltration membrane to 369.7 L·m^−2^·h^−1^ for the M20 ultrafiltration membrane. With the incorporation of a certain amount of PMO-MGs, the hydrophilicity of the resultant PMO-MGs/PES ultrafiltration membranes was enhanced, as revealed via dynamic contact angle measurement. Furthermore, the incorporation of microgel resulted in the creation of pores with a greater diameter and enhanced connectivity between pores. These two reasons that might account for the increase in the pure water flux for the M10, M15 and M20 ultrafiltration membranes.

The pure water flux of the M00 ultrafiltration membranes was slightly affected by the temperature of the water. Upon increasing the temperature of water, it slightly increased, which was mainly attributed to the decrease in viscosity and the increase in the mass transfer coefficient of the water at higher temperature. However, for the M05, M10, M15 and M20 ultrafiltration membranes that contained PMO-MGs, the pure water flux of the membranes significantly increased upon increasing the measured temperature. To quantify the thermo-sensitivity of pure water flux for PMO-MGs/PES composited ultrafiltration membranes, the ratio of the membranes’ pure water flux at 45 and 20 °C was identified as the thermo-sensitive coefficient R_45/20_. Figure 7B shows the pure water flux at 20 and 45 °C and the corresponding R_45/20_ values for the M00, M05, M10, M15 and M20 ultrafiltration membranes. It can be seen that for the M20 ultrafiltration membrane, the pure water fluxes at 20 and 45 °C were 369.7 and 618.7 L·m^−2^·h^−1^, respectively. The thermo-sensitive coefficient R_45/20_ was about 1.67, which indicated that the pure water flux of the M20 ultrafiltration membrane increased by 1.67 times when the temperature increased from 20 to 45 °C. The PMO-MGs shrunk as the temperature increased. Consequently, the membrane pore size increased, enhancing the water permeability of the ultrafiltration membrane and promoting the transfer of water. These results indicated that the incorporation of PMO-MGs gave the water permeation of PMO-MGs/PES composited ultrafiltration membranes a thermo-sensitive character.

### 3.7. BSA Rejection Property of PMO-MGs/PES Composited Ultrafiltration Membranes

**Figure 8:**
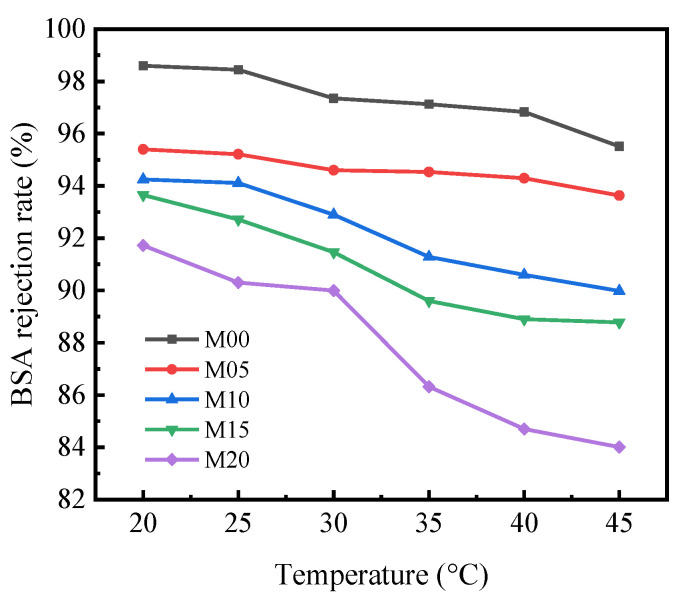
BSA rejection rate of PMO-MGs/PES composited ultrafiltration membranes.

The BSA rejection rate *R* of PMO-MGs/PES composited ultrafiltration membrane presented the rejection ability of the membrane to BSA under given pressure. Figure 8 shows the BSA rejection rate *R* of the M00, M05, M10, M15 and M20 ultrafiltration membranes at various temperatures with a pressure of 0.2 MPa. The *R* value was closely correlated with the surface hydrophilicity and pore structure of the ultrafiltration membranes. *R* of PMO-MGs/PES ultrafiltration membranes decreased with an increase in microgel content. The *R* values of the M00, M05, M10, M15 and M20 ultrafiltration membranes at 20 °C were 98.6%, 95.4%, 94.2%, 93.7% and 91.7%, respectively. As the temperature rose from 20 to 45 °C, the PMO-MGs shrunk, meaning that the pore size and the connectivity between the pores of the membranes increased. As a consequence, the *R* value of the corresponding ultrafiltration membranes decreased at higher temperatures. The *R* value of the M00 ultrafiltration membrane was about 95.5–98.6%, while the *R* value of the M20 ultrafiltration membrane was about 84.0–91.7% between 20 and 45 °C.

### 3.8. Antifouling Performance of PMO-MGs/PES Composited Ultrafiltration Membranes

**Figure 9:**
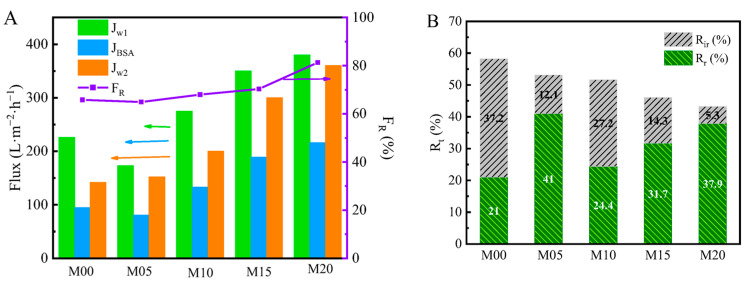
(**A**) Permeability flux and relative flux recovery ratio (*F_R_*) and (**B**) fouling resistance ratio of PMO-MGs/PES composited ultrafiltration membranes, purple line in (**A**): *F_R_*.

The flux recovery ratio (*F_R_*) was used to describe the anti-pollution characteristics of ultrafiltration membranes. As shown in Figure 9A, the *F*_R_ values of the M00, M05, M10, M15 and M20 ultrafiltration membranes were about 65.8%, 64.9%, 68.0%, 70.3% and 81.3%, respectively. It can be seen that BSA did not easily adhere to the surfaces of PMO-MGs/PES composited ultrafiltration membranes, and the antifouling performance of the membranes was enhanced. The hydrophilicity of composited ultrafiltration membranes was increased via the addition of PMO-MGs, and a hydration layer may have formed on the membrane’s surfaces, reducing the adherence of proteins to the membrane’s surfaces. The composited ultrafiltration membranes could be more easily cleaned and, thus, had a higher flux recovery rate.

Three fouling resistance ratios, namely the total pollution (*R_t_*), reversible pollution (*R_r_*) and irreversible pollution (*R_ir_*) ratios, were used to quantify the antifouling performance of PMO-MGs/PES composited ultrafiltration membranes, as shown in Figure 9B. *R_t_* decreased from 58.2% for the M00 ultrafiltration membrane to 43.2% for the M20 ultrafiltration membrane, showing that the incorporation of PMO-MGs provided better pollution resistance for the composited ultrafiltration membranes. Similarly, *R_ir_* and *R_t_* decreased as the content of PMO-MGs in the ultrafiltration membranes increased. These results showed that adding PMO microgels to the composited ultrafiltration membranes increased their antifouling performance. Furthermore, after performing the cleaning processes and measurements of water filtration several times, the composited membranes were not damaged and could be used in a normal manner. The mechanical properties of the composited membranes could meet the requirements of ultrafiltration operation.

## 4. Conclusions

PMO-MGs/PES composited ultrafiltration membranes were successfully prepared via the NIPS method and used PMO-MGs and PES mixed solution as their casting solution. PMO-MGs were mainly located on the channel surface of the membranes. At 20 °C, the pure water flux increased to 369.7 L·m^−2^·h^−1^ for M20 ultrafiltration membranes, and the BSA rejection ratio was 91.7%, indicating that the presence of PMO-MGs significantly improved the surface hydrophilicity and antifouling performance of the composited membranes. The incorporation of PMO-MGs also gave the composited membranes a thermo-sensitive character. When we increased the temperature from 20 to 45 °C, the pure water flux of the M20 ultrafiltration membranes significantly increased from 369.7 to 618.7 L·m^−2^·h^−1^.

## Figures and Tables

**Table 1:** The compositions of casting solutions used to prepare PMO-MGs/PES composited ultrafiltration membranes.

Membrane Code	PMO Microgels (g)	PES (g)	NMP (g)	MG/PES (wt.%)
M00	0.0	18	100	0
M05	0.9	18	100	5
M10	1.8	18	100	10
M15	2.7	18	100	15
M20	3.6	18	100	20

**Table 2:** Surface atomic concentration (%) and atomic ratio of M00 and M20 ultrafiltration membranes.

Membrane Code	Atomic Concentration (%)				Atomic Ratio		
	C	O	N	S	O/C	N/C	N/O
M00	79.02	15.16	0	5.82	0.192	0	0
M20	71.71	21.99	2.99	3.30	0.307	0.042	0.136

**Table 3:** Porosity and pore size of M00, M05, M10, M15 and M20 ultrafiltration membranes.

Membrane Code	Porosity, *ε* (%)	Pore Size, *r_m_* (nm)
M00	74	18
M05	89	12
M10	73	21
M15	70	27
M20	66	32

## Data Availability

Data sharing is not applicable to this article.
